# Developing International Classification of Disease code definitions for the study of enteric infection sequelae in Canada

**DOI:** 10.14745/ccdr.v49i78a01

**Published:** 2023-08-01

**Authors:** Eleni Galanis, Azita Goshtasebi, Yuen Wai Hung, Jonathan Chan, Douglas Matsell, Kristine Chapman, Gilaad Kaplan, David Patrick, Bei Yuan Zhang, Marsha Taylor, Dimitra Panagiotoglou, Shannon Majowicz

**Affiliations:** 1 Faculty of Medicine, University of British Columbia, Vancouver, BC; 2 British Columbia Centre for Disease Control, Vancouver, BC; 3 School of Public Health Sciences, University of Waterloo, Waterloo, ON; 4 Departments of Medicine & Community Health Sciences, University of Calgary, Calgary, AB; 5 Department of Epidemiology, Biostatistics and Occupational Health, McGill University, Montréal, QC

**Keywords:** enteric infections, sequelae, administrative data, case definition

## Abstract

**Background:**

Enteric infections and their chronic sequelae are a major cause of disability and death. Despite the increasing use of administrative health data in measuring the burden of chronic diseases in the population, there is a lack of validated International Classification of Disease (ICD) code-based case definitions, particularly in the Canadian context. Our objective was to validate ICD code definitions for sequelae of enteric infections in Canada: acute kidney injury (AKI); hemolytic uremic syndrome (HUS); thrombotic thrombocytopenic purpura (TTP); Guillain-Barré syndrome/Miller-Fisher syndrome (GBS/MFS); chronic inflammatory demyelinating polyneuropathy (CIDP); ankylosing spondylitis (AS); reactive arthritis; anterior uveitis; Crohn’s disease, ulcerative colitis, celiac disease, erythema nodosum (EN); neonatal listeriosis (NL); and Graves’ disease (GD).

**Methods:**

We used a multi-step approach by conducting a literature review to identify existing validated definitions, a clinician assessment of the validated definitions, a chart review to verify proposed definitions and a final clinician review. We measured the sensitivity and positive predictive value (PPV) of proposed definitions.

**Results:**

Forty studies met inclusion criteria. We identified validated definitions for 12 sequelae; clinicians developed three (EN, NL, GD). We reviewed 181 charts for 6 sequelae (AKI, HUS, TTP, GBS/MFS, CIDP, AS). Sensitivity (42.8%–100%) and PPV (63.6%–100%) of ICD code definitions varied. Six definitions were modified by clinicians following the chart review (AKI, TTP, GBS/MFS, CIDP, AS, reactive arthritis) to reflect coding practices, increase specificity or sensitivity, and address logistical constraints.

**Conclusion:**

The multi-step design to derive ICD code definitions provided flexibility to identify existing definitions, to improve their sensitivity and PPV and adapt them to the Canadian context.

## Introduction

Enteric infections are a major cause of disability and death globally and in Canada ((1–3)). In addition to the acute gastrointestinal manifestations, enteric infections can also lead to sequelae such as hemolytic uremic syndrome (HUS), inflammatory bowel disease, and Guillain-Barré Syndrome (GBS) ((4–6)). In separate but related work, we are conducting a retrospective population-based cohort study to determine the likelihood of developing sequelae following enteric infections, as well as their burden of illness and cost, in British Columbia (BC), Canada ((7)). To do so, we require International Classification of Diseases (ICD) code definitions for the sequelae of interest.

Despite the increasing use of administrative health data in epidemiological research, there is a lack of verified ICD code-based case definitions broadly ((8)), and a lack of validated definitions for most enteric infection sequelae in the Canadian context. Our objective here was to identify, or develop and validate, ICD code-based case definitions for 15 sequelae of enteric infections (**Table 1**).

**Table 1:** Administrative case definitions for sequelae of enteric infections, showing International Classification of Diseases codes identified by study stage

Sequela	ICD codes identified, by study stage^a^			Final definition^a^	
	Literature review	Recommendation by clinical experts	Chart review	ICD codes	Timeframe
Acute kidney injury	ICD-9: 584 ICD-10: N17, Z99.2	ICD-9: 584 ICD-10: **N17.9**	ICD-9: N/A ICD-10: N17.9, **N17.8, N17.0**	ICD-9: 584 ICD-10: N17.0, N17.8, N17.9	One or more hospitalizations
Hemolytic uremic syndrome	ICD-9: 283.11 ICD-10: D59.3	ICD-9: 283.11 ICD-10: D59.3	ICD-9: N/A ICD-10: D59.3	ICD-9: 283.11 ICD-10: D59.3	Either (or both of): (a) one or more hospitalizations OR (b) more than two physician claims within a span of two years
Thrombotic thrombocytopenic purpura (misdiagnosed hemolytic uremic syndrome)^b^	ICD-9: 287.31, 287.33 ICD-10: M31.1, N08.5	ICD-9: **287.3** ICD-10: M31.1, N08.5	ICD-9: N/A ICD-10: M31.1, N08.5	ICD-9: **287,** 287.3 ICD-10: M31.1, N08.5 Together with: **ICD-9: 584** **ICD-10: N17.0, N17.8, N17.9**	Either (or both of): (a) one or more hospitalizations OR (b) one or more physician claims Together with: one or more hospitalizations or physician claims with an ICD code for acute kidney injury (see above) in the week before or after
Guillain-Barré syndrome/Miller Fisher syndrome	ICD-9: 357 ICD-10: G61.0	ICD-9: 357, **356+IVIG** ICD-10: G61.0	ICD-9: 356 ICD-10: G61.0	ICD-9: **356**, **357** ICD-10: G61.0	Either (or both of): (a) one or more hospitalizations OR (b) two or more physician claims within a span of fewer than three months
Chronic inflammatory demyelinating polyneuropathy	ICD-9: 357.81 ICD-10: G61.8	ICD-9: 357.81, **356+IVIG** ICD-10: G61.8	ICD-9: **357**, 356+IVIG ICD-10: G61.8	ICD-9: **356**, **357.81** ICD-10: G61.8	Either (or both of): (a) one or more hospitalizations OR (b) two or more physician claims occurring three or more months apart
Ankylosing spondylitis	ICD-9: 720 ICD-10: M45	ICD-9: 720, **720.0, 720.8, 720.9** ICD-10: M45	ICD-9: 720, 720.0 ICD-10: M45	ICD-9: 720, 720.0 ICD-10: M45	Either (or both of): (a) one or more hospitalizations OR (b) two or more physician claims within a span of two or fewer years
Reactive arthritis	ICD-9: 711 ICD-10: N/A	ICD-9: 711, **696, 714** ICD-10: M02	N/A	ICD-9: 711, 696, 714 ICD-10: M02	Two or more physician claims that are both: (a) two or more months apart AND (b) within a span of five or fewer years
Anterior uveitis	ICD-9: 364 ICD-10: H20.0	ICD-9: 364 ICD-10: H20.0	N/A	ICD-9: 364 ICD-10: H20.0	Either (or both of): (a) one or more hospitalizations OR (b) one or more physician claims
Crohn’s disease	ICD-9: 555 ICD-10: K50	ICD-9: 555 ICD-10: K50	N/A	ICD-9: 555 ICD-10: K50	Either (or both of): (a) two or more hospitalizations OR (b) four or more physician claims within a span of two years
Ulcerative colitis	ICD-9: 556 ICD-10: K51	ICD-9: 556 ICD-10: K51	N/A	ICD-9: 556 ICD-10: K51	Either (or both of): (a) two or more hospitalizations OR (b) four or more physician claims within a span of two years
Irritable bowel syndrome	ICD-9: 564.1 ICD-10: K58.0, K58.9, F45.3	ICD-9: 564.1 ICD-10: K58.0, K58.9, F45.3	N/A	ICD-9: 564.1 ICD-10: K58.0, K58.9, F45.3	One or more physician claims or hospitalizations AND Either: (a) no Crohn’s disease, ulcerative colitis or celiac disease OR (b) a second claim more than six months apart
Celiac disease	ICD-9: 579 ICD-10: K90.0	ICD-9: 579 ICD-10: K90.0	N/A	ICD-9: 579 ICD-10: K90.0	Either (or both of): (a) one or more hospitalizations OR (b) one or more physician claims
Erythema nodosum	-	ICD-9: 695.2, 729.3 ICD-10: L52, M79.3	N/A	ICD-9: 695.2, 729.3 ICD-10: L52, M79.3	Either (or both of): (a) one or more hospitalizations OR (b) one or more physician claims
Neonatal listeriosis	-	ICD-9: 771.2 ICD-10: P37.2	N/A	ICD-9: 771.2 ICD-10: P37.2	One or more hospitalizations
Graves’ disease	-	ICD-9: 242.0, 242.01, 242.91, 242.9 ICD-10: E05.0, E05.90, E05.91	N/A	ICD-9: 242.0, 242.01, 242.91 ICD-10: E05.0	Either (or both of): (a) one or more hospitalizations OR (b) one or more physician claims

Abbreviations: ICD, International Classification of Diseases; N/A, not applicable

^a^ Bolded text shows additions/changes at each stage of case definition development

^b^ Thrombotic thrombocytopenic purpura is not a sequela of enteric infection; this definition was developed to capture historical misdiagnosis of hemolytic uremic syndrome as thrombotic thrombocytopenic purpura

## Methods

We used a multi-step process to identify case definitions for the 15 sequelae (Table 1).

### Literature review, clinician assessment

We searched MEDLINE (1946–July 2018) and EMBASE (1974–July 2018) databases for peer-reviewed studies published in English or French using the following terms: [([administrative OR hospital discharge OR health service OR physician] AND [data OR claim* OR record* OR database*]) OR (case definition* OR ICD-9 OR ICD-10 OR international classification of diseases)]; AND [(validity OR validate* OR validation OR agreement OR accuracy OR sensitivity OR specificity or predictive value)] AND [(search terms for sequelae of interest, as listed in **Supplemental material, Table S1**)].

We included studies with a case definition based on ICD-9 or ICD-10 coding of one or more of the sequelae validated against a gold standard that revealed at least one measure of validity (sensitivity, specificity, positive predictive value [PPV], or negative predictive value). Studies were evaluated for eligibility, independently through title and abstract screening, and those that met the eligibility criteria underwent full text review. Disagreements were resolved by discussion and consensus. Where we identified multiple case definitions for a sequela, we selected those that were validated in Canada to ensure comparable coding practices or, if Canadian studies were not available, those with the highest measures of validity. Where we identified no relevant studies, clinicians proposed ICD-based case definitions based on expert opinion.

We invited clinician specialists in rheumatology, neurology, nephrology and gastroenterology with expertise in the sequelae of interest and based in BC or Alberta to participate in the study. They reviewed the case definitions from the literature review and revised them to reflect BC or Canadian coding practices.

### Medical chart review

#### Setting, data sources, and ethics

We reviewed patients’ charts from four tertiary care centres in Vancouver, BC, during the fall of 2018. Centres were selected based on the following criteria: most likely to see patients with sequelae of interest and sufficient numbers to meet sample size. Centres most likely to see the sequelae were selected based on whether the condition was more likely to be assessed in inpatient (e.g. acute kidney injury, AKI) or outpatient (e.g. ankylosing spondylitis, AS) settings and the age at which the condition is most likely to occur (e.g. thrombotic thrombocytopenic purpura [TTP] occurs mainly in adults and HUS, mainly in children). Vancouver Coastal Health and the British Columbia Children’s Hospital granted operational approvals to access and review patient charts.

We conducted chart reviews for the following six sequelae: AKI, HUS, TTP, GBS/Miller-Fisher syndrome (MFS), AS, and chronic inflammatory demyelinating polyneuropathy (CIDP). Eligible charts included those for patients with at least one admission or visit between January 1, 2003, and December 31, 2016. We tailored our chart sampling strategy by sequelae and centre (**Table 2**), to accommodate facility differences. Chart reviews were not conducted for the other nine sequelae for the following reasons: 1) validated Canadian case definitions already existed (for Crohn’s disease and ulcerative colitis); 2) clinical experts deemed further data were not required (for neonatal listeriosis [NL]); or 3) charts were not readily available (all other sequelae).

**Table 2:** Patient charts and sequelae reviewed, by medical centre

Centre	Number of charts reviewed (% of all charts reviewed)	Sequelae assessed (Number of charts reviewed)	Patient registry	Type of charts reviewed	Source of ICD codes
Paediatric tertiary care hospital	20 (11%)	AKI (n=11) HUS (n=9)	Yes	Both electronic and paper	Hospital health records database
Adult tertiary care hospital	107 (59%)	AKI (n=31) HUS (n=11) TTP (n=14) GBS (n=26) MFS (n=3) CIDP (n=11) AS (n=11)	No	Both electronic and paper	Hospital health records database
Adult neurology referral clinic	27 (15%)	GBS (n=9) MFS (n=5) CIDP (n=13)	Yes	Electronic only	Electronic medical record
Adult rheumatology referral clinic	27 (15%)	AS (n=27)	Yes	Electronic only	Electronic medical record

Abbreviations: AKI, acute kidney injury; AS, ankylosing spondylitis; CIDP, chronic inflammatory demyelinating polyneuropathy; GBS, Guillain-Barré syndrome; HUS, hemolytic uremic syndrome; ICD, International Classification of Diseases; MFS, Miller-Fischer syndrome; TTP, thrombotic thrombocytopenic purpura

In the paediatric hospital, a randomized sample of patients with AKI and HUS were selected from a registry maintained by the nephrology department. Charts were reviewed, and ICD codes were retrieved from the health records database (Table 2). In the adult hospital, the Health Records Department converted the list of sequelae into ICD codes; a random sample of charts with one of these ICD codes for a given admission was reviewed. In the neurology clinic, charts were identified by the neurology co-author; we reviewed all MFS patients’ charts, and a convenience sample of charts for patients with GBS or CIPD. In the rheumatology clinic, a convenience sample of charts from patients with AS was selected and reviewed from a registry maintained by the rheumatology co-author.

### Diagnosis verification and data abstraction

We compared each ICD code against clinical criteria and/or the physician diagnosis. For AS, we used rheumatologist diagnosis as the gold standard as no diagnostic criteria existed. We developed clinical criteria (see Supplemental material, **Table S2**) using a literature review and clinical expert opinion, and created data abstraction forms for renal (AKI, TTP, HUS), neurological (GBS/MFS, CIDP), and rheumatologic (AS) sequelae. We piloted the forms with 4–5 charts each and included the pilot data in the final analysis.

A medical chart abstractor reviewed the first visit/admission for each ICD code of interest. If the criteria were not met or diagnosis was not confirmed, the next visit/admission was reviewed. The abstractor evaluated all visits/admissions with the same ICD code or sequelae and abstracted all ICD codes recorded for the visit/admission and the diagnosis made by the attending physician on the discharge and/or consult note.

### Analysis

We assessed agreement between the ICD codes and clinical criteria or physician diagnosis by calculating sensitivity (for the two clinics and paediatric hospital, where patients were identified based on diagnosis), and PPV (for the adult hospital, where patients were identified by ICD code), with 95% confidence intervals.

We developed case definitions using ICD-9 and ICD-10 codes given that during the study period (2005–2014) in BC, ICD-9 codes were used for physician billings and ICD-10 codes were used by hospitals.

### Finalizing the case definitions

Clinicians with expertise in rheumatology, neurology, nephrology, gastroenterology and hematology reviewed the definitions resulting from the above steps to generate final definitions.

## Results

### Literature review and clinician assessment

Our search returned 1,414 articles; of which 39 met the inclusion criteria ((9–47)). One additional article, not uncovered through the search but meeting our eligibility criteria, was identified by a co-author for a total of 40 articles ((48)). For three sequelae (erythema nodosum [EN], NL and Graves’ disease [GD]), no articles met our search criteria. Of the 40 articles, there were six from Canada, covering AS, ulcerative colitis, Crohn’s disease, and celiac disease. Details on the 40 articles are in the Supplemental material, **Table S3**.

From these 40 articles, we derived initial case definitions for 12 sequelae (Table 1). Clinicians reviewed these and made minor changes to AKI, TTP, GBS/MFS, CIDP, AS and reactive arthritis to represent coding practices in BC. The use of intravenous immunoglobulin therapy was added to help identify cases of GBS/MFS and CIDP. For the three sequelae for which no articles were identified (EN, NL, GD), case definitions were proposed by clinicians (Table 1).

### Medical chart review

We reviewed 181 charts from four medical centres (Table 2).

The agreement between the clinical criteria and physician diagnosis and the corresponding ICD codes is presented in **Table 3**. Sensitivity of the proposed AKI ICD codes was low (42.8%–44.4%), while sensitivity of the proposed ICD codes for HUS, GBS, MFS, CIDP and AS was high (85.7%–100%). The PPV varied by sequelae and reference standard, from 63.6% for AS to 100% for HUS, TTP and MFS.

**Table 3:** Sensitivities and positive predictive values (with 95% confidence intervals) of International Classification of Diseases codes

Sequela	Sensitivity				Positive predictive value			
	ICD codes	Number of charts with sequela	Reference standard		ICD codes	Number of charts with ICD codes	Reference standard	
			Clinical criteria (95% CI)	Physician diagnosis (95% CI)			Clinical criteria (95% CI)	Physician diagnosis (95% CI)
Acute kidney injury	N17.0, N17.8, N17.9	11	44.4% (13.7–78.8)	42.9% (9.9–81.6)	N17.0, N17.8, N17.9	31	100.0% (89.0–100.0)	80.6% (63.7–90.8)
Hemolytic uremic syndrome	D593	9	100.0% (54.1–100.0)	85.7% (42.1–99.6)	D593	11	90.9% (62.3–98.4)	100% (74.1–100.0)
Thrombotic thrombocytopenic purpura	M31.1, N08.5	0	-^a^	-^a^	M31.1	14	100% (78.5–100.0)	100% (78.5–100.0)
Guillain-Barré syndrome	356	9	100.0% (63.4–100.0)	100.0% (63.4–100.0)	G610	26	68.0% (48.4–82.8)	92.5% (75.0–97.8)
Miller Fisher syndrome	356	5	100.0% (39.8–100.0)	100% (39.8–100.0)	G610	3	33.3% (6.1–79.2)	100% (43.9–100.0)
Chronic inflammatory demyelinating polyneuropathy	356, 357	13	100.0% (71.5–100.0)	100% (71.5–100.0)	G618	11	70% (39.7–89.2)	80% (49.0–94.3)
Ankylosing spondylitis	720	27	-^a^	92.5% (75.7–99.1)	M45	11	-^a^	63.6% (35.4–84.8)

Abbreviations: CI, confidence interval; ICD, International Classification of Diseases; -, no results are presented

^a^ Charts identified using ICD codes, thus only positive predictive value could be calculated

### Final administrative case definitions

Clinicians reviewed the chart review findings and provided final revisions and approval of case definitions (Table 1). Given that TTP was included in the study as a proxy for HUS, we combined codes for TTP and AKI to increase PPV. Given that the subsequent cohort study does not have access to intravenous immunoglobulin data, this was dropped from the GBS/MFS and CIDP definitions.

## Discussion

This study identified, developed and validated ICD-based case definitions for 15 sequelae of enteric infections. These are now being used in a population-wide cohort study to determine the likelihood of developing sequelae following enteric infections and their burden in terms of illness and cost ((7)).

We used a multi-method approach that combined 1) a literature review, 2) clinician consultation, 3) chart reviews and 4) final clinician consultation to generate valid case definitions relevant to our study context, and we documented how each of these methods affected the final case definitions. This multi-method explicit approach is not common; most studies derive case definitions solely using medical chart reviews.

Six (HUS, AKI, TTP, GBS/MFS, CIDP, AS) conditions underwent all four steps (Table 1). Only the HUS definition underwent no changes from the initial literature review. Of the remaining five, all were slightly modified based on clinician input and three (AKI, GBS/MFS, CIDP) were further amended following the chart review. Of the nine conditions that did not undergo a chart review, only the reactive arthritis definition was modified based on clinician input. Five case definitions identified in the literature (anterior uveitis, Crohn’s disease, ulcerative colitis, irritable bowel syndrome, celiac disease) remained unchanged throughout and three (EN, NL, GD) were entirely developed by clinicians.

Some of the clinician changes were made to reflect coding practices by hospitals (e.g. use of M79.3 for EN) or clinicians (e.g. use of 287 rather than 287.31 for TTP) in BC. Other changes were made to increase PPV (e.g. change from N17 to N17.0/8/9 for AKI) or sensitivity (e.g. add 696 and 714 for reactive arthritis). A final review addressed logistical constraints (e.g. the planned cohort study cannot assess intravenous immunoglobulin administration).

The findings from our chart review were varied. Sensitivity of the proposed case definitions was generally as high as, or higher than, that reported by others for the same or similar ICD codes ((12,15,16,18,24–27)). Exceptionally, we found low sensitivity (42.8%–44.4%) for AKI. Given that all our patients were selected from an AKI registry, we believe that they had AKI but were missing an AKI ICD code. Among those without an AKI code, five patients were admitted for other reasons and developed AKI while in hospital and two patients were coded as having chronic kidney injury rather than AKI. Interestingly, others also found low sensitivity for AKI-related ICD codes ((9)).

The PPV of our proposed case definitions was also generally as high as, or higher than the PPV reported by others ((9,15,16)). The chart review identified the use of two additional codes for AKI in hospitalized adults: N17.0, N17.9. The addition of these codes increased the PPV from 60.0% to 80.6%. The PPV of AS (63.6%) was lower than the one found by other studies ((24,25,28)); we found that four of 11 hospitalizations coded as M45 were for patients diagnosed with rheumatoid arthritis, not AS. The PPV of code G610 for MFS based on clinical criteria was low (33.3%) due to the lack of nerve conduction study results in two patient charts; however, all three patients were clinically diagnosed with MFS.

The main challenge in planning the chart review was determining the sample size to accurately estimate sensitivity and specificity and be representative of local coding practices. The literature on the ideal sample size to assess sensitivity and PPV is limited; authors typically review all charts within a period or at a given site ((16)). We decided to treat this as an exploratory or descriptive study where authors suggest 10–20 charts per question or variable of interest ((49)). We aimed for a minimum of 10 charts per sequelae, which seemed reasonable given the rarity of some of the conditions, the resources we had and the homogeneity of ICD coding in most instances.

## Strengths and limitations

We conducted a chart review for only six of the 15 enteric infection sequelae. These six were selected based on clinician recommendations and because charts for these conditions were readily available. This convenience sample may not be entirely representative of coding practices across our entire study area; however, patients from BC who have the reviewed conditions are mostly cared for in the tertiary care centres included in the study. The sensitivity and PPV calculations were limited by a number of factors. For patients identified through a registry, if only a subset of their charts was reviewed, the code of interest may not be apparent. Clinical data to confirm a diagnosis may be incomplete or absent because patients were transferred from other hospitals or assessed in other settings. For some conditions (e.g. irritable bowel syndrome), the wide spectrum of illness and the large number of health care providers who encountered these patients likely lead to ICD-coding variability. For some conditions, multiple validated case definitions exist (e.g. inflammatory bowel disease) and we had to select among them ((30,33,35,50)).

Despite these limitations, there was relatively good concordance in ICD codes between the four methods used—the changes we made to our case definitions were minor and the final definitions were very similar to those validated and used by other researchers. The main concern is the low PPV for ICD code M45, which will identify a substantial number of non-AS hospitalizations, and the low sensitivity of AKI codes, which will underestimate the number of AKI events. These issues need to be accounted for in our future analyses. Our approach allowed us to verify codes identified in the literature with local practices and local chart review validation and benefited from the knowledge of local clinicians.

## Conclusion

The multi-step design to derive ICD-code based case definitions allowed us to identify previously validated definitions to adapt them to our study context, and to develop and validate definitions using clinical expertise and medical chart reviews. These findings will support future analyses to determine the likelihood, burden and cost of developing sequelae following enteric infections. They also provide Canadian researchers with validated ICD code definitions for 15 chronic conditions.

## Supplemental material

These documents can be accessed on the Supplemental material file.
Table S1: Search terms used in the literature review to identify International Classification of Diseases code-based case definitions for enteric infection sequelae
Table S2: Sequelae assessed during the chart review and their clinical criteria
Table S3: Results of the literature review to identify International Classification of Diseases code-based case definitions for enteric infection sequelae
